# Outcome of thyroid associated ophthalmopathy treated by radiation therapy

**DOI:** 10.1186/1748-717X-6-46

**Published:** 2011-05-13

**Authors:** Mirna Abboud, Asma Arabi, Ibrahim Salti, Fady Geara

**Affiliations:** 1 Department of Radiation Oncology, American University of Beirut Medical Center, Beirut, Lebanon; 2 Division of Endocrinology and Metabolism, American University of Beirut Medical Center, Beirut, Lebanon

## Abstract

Thyroid associated orbitopathy is a common manifestation of Graves disease. Many options can be considered for treatment. In this case series, we reviewed the medical records of 17 patients who received radiation therapy (RT) for GO in a tertiary care center between 1997 and 2007. All patients received 20 Gy to both orbits and 12 of them (71%) had already received one or more trials of steroid therapy prior to RT. After a median follow-up of 2 years, a subjective improvement in exophthalmos and vision was reported by all patients at the end of RT but only 3 patients reported a decrease in their diplopia immediately after therapy. Symptoms continued to improve with time in many patients: 22% had complete reversal of their symptoms and signs, and the remaining 78% had partial improvement. Two patients developed recurrent signs and symptoms, both of them were smokers who continued to smoke after treatment. About 60-65% of patients responded favorably to RT alone which increased to 87-97% when RT is combined with steroids. No patients developed late toxicity during the follow-up period. We conclude that RT is an effective treatment option in GO even in patients who failed previous treatment with steroids or surgical decompression. Based on our own clinical experiences and the literature data, the combination of RT and intravenous corticosteroid administration may improve the response rate.

## Introduction

Thyroid associated orbitopathy [TAO], is the most common extrathyroidal manifestation of Graves disease, and continues to be a dilemma with regard to its pathogenesis and its therapy.

Clinically evident ophthalmopathy occurs in 10% to 25% of patients with Graves thyrotoxicosis. This prevalence may increase up to 45% if eyelid changes are included, and eye involvement detected by computed tomography (CT) are considered [1,2]. Glucocorticoids have been used for the treatment of TAO for more than 40 years [3] but their effect in decreasing proptosis and improving ocular motility has not always been impressive and ranges between 40 and 80% [4-6]. On the other hand, orbital radiotherapy has been used for treatment of TAO for more than sixty years, and this treatment methodology has become widely used over the last years, in view of its nonspecific antiinflammatory effect and the high radiosensitivity of lymphocytes infiltrating the orbital space [7]. However, the precise therapeutic target in the treatment of radiotherapy in TAO is still not well understood. It remains unclear whether lymphocytes, mesenchymal cells or the combination of both are the target tissue. Clinical studies have shown that the effects of orbital irradiation may take several weeks to become manifest but are more prolonged than those of glucocorticoids. Although TAO can sometimes be controlled in the long term with orbital irradiation alone [8], the reduction in proptosis and the improvement in ocular motility, are not impressive [9].

To our knowledge, the effect of RT on TAO has not been assessed in the Middle East region. In this retrospective study, we reviewed the charts of patients who were treated for TAO with radiation therapy at our tertiary care center in the Middle East between 1997 and 2007. Seventeen patients were available for this study. Baseline characteristics of the patients, their presenting symptoms, biochemical data, additional treatment and outcome data after radiation therapy were collected. The findings were compared and contrasted to those reported in the literature.

## Patients and methods

Our research was in compliance with the Helsinki Declaration and approval was obtained from the IRB committee at our institution. All patients gave their informed consent to partake in the project. Seventeen patients (8 men and 9 women) were identified during the study period (Table 1). The median age was 50 years (28-67) in men and 49 years (20-56) in women. The majority had the onset of hyperthyroidism before GO (72%), whereas GO was the presenting manifestation in four patients only. The median interval between the onset of hyperthyroidism and the onset of GO was 6 months (0-120). The median TSH, free T3 and free T4 before starting radiotherapy were 0.16 μU/ml, 2.5 ng/dl and 1.5 ng/dl respectively. Four patients were hyperthyroid and 13 were euthyroid. The median duration of GO prior to receiving RT was 16 months (1 month-8 years). Twelve patients received radioactive iodine (RAI) at a median interval of 42 months (24-216) before developing GO. Only 1 patient received RAI after developing GO. Twelve patients were smokers. All patients had proptosis. Palpebral edema was present in 10 patients (59%) and ophthalmoplegia in 11 (65%). Symptoms were bilateral in two thirds of affected patients and unilateral in one third of them.

**Table 1:** Baseline characteristics of the study population

Characteristic	Number (Percentage %)
**Gender**	
**Male**	8 (47)
**Female**	9 (53)
Age* (years)	50 (20-67)
**Hyperthyroidism**	
**Yes**	16 (94.1)
**No**	1 (5.9)
**Smoking**	
**Current**	10 (71.4)
**Ex**	2 (14.3)
**No**	2 (14.3)
**Prior Radioactive Iodine**	
**Yes**	13 (76.5)
**No**	4 (23.5)
**Prior steroid therapy**	
**Yes**	12 (70.6)
**No**	5 (29.4)
**Prior Surgery**	
**Yes**	3 (17.6)
**No**	14 (82.4)
**Interval between**	6 [0-120]
**hyperthyroidism and**	
Ophtalmopathy* (months)	
**Interval between**	68 [24-216]
**Radioiodine and Radiation**	
Therapy* (months)	

*Values are Median [min-max]

All 17 patients were treated by orbital radiation, with concurrent steroids (except in one patient) mainly consisting of high dose intravenous methylprednisolone (1 g/day for 5 days). Twelve patients (71%) had also received one or several courses of steroids prior to RT with variable regimen ranging from 1 mg/kg/day of oral prednisone with gradual tapering, to 1 mg/day of IV methylprednisolone over 5 days. Three patients had undergone bilateral surgical orbital decompression at an interval of 2 months to 1 year prior to RT but they were referred for radiation therapy because of persistence of their symptoms, consisting mainly of exophthalmos and poor vision. All patients received a dose of 20 Gy to both eyes fractionated in 10 daily doses over a 2-week period. Treatment was delivered thru parallel opposed 6MV beams using a downward 5° tilt to avoid direct irradiation to the contralateral lens. The treated volume consisted of the entire orbital content from the orbital apex posteriorly to the fleshy cantus anteriorly. Patients were followed at regular interval by their endocrinologist and opthtalmologist. Subjective assessment of diplopia and exophthalmos were performed at baseline, at the end of radiotherapy and at the last visit. Evaluation immediately after radiation therapy is available for all patients but long term follow-up is available for only 9 patients. Median follow-up time was two years.

## Results

### Immediate response

Sixteen patients (94.1%) described subjective improvement in their vision and a decrease in the exophthalmos at the end of RT. Eight patients (47%) had no change in their diplopia the last day of RT and 3 reported some improvement. These outcomes are detailed in Table 2. In terms of acute toxicity, two patients developed bilateral eye redness toward the end of RT.

**Table 2:** Outcome of radiation therapy

Outcome	Number (Percentage)
**Overall outcome**	
Stable	1 (5.9%)
Improved	16 (94.1%)

**Diplopia**	
Stable	8 (72.7%)
Improved	3 (27.3%)

**Exophtalmos**	
Stable	1 (5.9%)
Improved	16 (94.1%)

### Long term follow up

Data on long term effect are available for 9 patients (53%). 2 among them (22%) had complete reversal of their diplopia and exophthalmos which were maintained up to 3 year follow-up, whereas 7 among them (78%) had partial response only in exophthalmos, diplopia or both. These 7 cases are detailed as follows:

- Residual minimal diplopia involving vertical gaze only persisted in one patient after six months, and in another patient 4 years after the end of RT.

- One patient had minimal response in her exophthalmos after 2 months of follow-up but she was later lost to follow up and further assessment was not available.

- Another patient had residual minimal exophthalmos after 2 years of follow-up.

- One patient had good results initially but her exophthalmos was still cosmetically unacceptable to her and underwent debulking orbital surgery one year after RT.

- The remaining two women experienced recurrence of their symptoms, both of them were smokers who did not quit smoking after RT. The first patient developed recurrence of diplopia and exophthalmos 5 months after RT. Her diplopia, but not the exophthalmos responded well to pulse steroids. The other patient developed bilateral eyelid swelling and hyperemia but without diplopia, two years following RT, and responded well to oral prednisone.

## Discussion

TAO is considered an autoimmune disorder and like many autoimmune diseases, it is more prevalent in women than in men [8]. The prevalence of GO is five times higher in women than in men [10]. The distribution is bimodal, with peak incidence in the age groups 40 to 44 years and 60 to 64 years in women and 45 to 49 years and 65 to 69 years in men. Thus, the median age in our study (49 and 50 years) reflects the general age prevalence. In our study, females were slightly more frequent than males. However, this does not reflect the true female to male ratio in our population, since we only included those referred for radiation therapy.

Many classification systems have been used for TAO including the NOSPECS classification of eye changes of Graves' disease: [N stands for No signs or symptoms; O, only signs, no symptoms; S, soft tissue involvement; P, proptosis; E, extraocular muscle involvement; C, corneal involvement; S, sight loss (due to optic nerve involvement)]. Information regarding antithyroid antibodies were lacking in our series. However, there is no established correlation between serum levels of antithyroid antibodies and severity of ophthalmopathy, although it has been reported that severe ophthalmopathy tends to develop in the presence of high titers in the serum antithyroid-stimulating antibody [11-13]. Once a patient has Graves disease, the major clinical risk factor for developing thyroid eye disease is smoking [10]. A MEDLINE search identified 25 studies on the association between smoking and thyroid diseases and found that patients with thyroid eye disease are four times more likely to be smokers or former smokers than never smokers [14]. The detrimental effect of smoking to the orbital immune process is mediated by modulating the release of autoantigens from the thyroid, inducing local hypoxia which increases glycosaminoglycan production by fibroblasts [15], and by modulating the secretion of cytokines and cytokine antagonists such as the IL-1 receptor antagonist [16]. The response rate to treatment modalities including steroids and retrobulbar irradiation is better in non smokers compared to smokers [17]. Moreover, smoking cessation might have a beneficial effect on GO even when the disease is already present. The role of smoking as an exacerbating factor was shown in our case series where 84% of patients have been exposed to tobacco smoking the majority being current smokers at the time of diagnosis and the two patients who continued to smoke after RT developed recurrence of their symptoms.

Five patients have already received radio-active-iodine at the time of RT, at an interval of 2 to 18 years before RT. Whereas antithyroid drugs or thyroid surgery do not seem to alter the natural course of Graves ophthalmopathy, ^131^I therapy carries a small risk for development or worsening of eye changes, which fortunately are mostly transient in nature [18,19]. This adverse effect of ^131^I therapy is particularly apparent in patients who smoke, who have a pretreatment serum triiodothyronine of > 5 nmol/l, high levels of serum anti-thyrotropin receptor antibodies, and the presence of ocular signs or symptoms [20]. Cigarette smoking was also shown to increase the risk of progression of ophthalmopathy after radioiodine therapy [21].

All our patients received a dose of 20 Gy to both eyes fractionated in 10 daily doses over a 2-week period. Orbital radiotherapy was utilized for many years for the treatment of Graves ophthalmopathy [7]. Nowadays, most centers use linear accelerators delivering 4-6 megavolts and a 4 × 4-cm lateral field slightly angled posteriorly to avoid as much as possible irradiation to the contralateral lens. The most common delivered dose is 20 Gy [7]. A lower dose of 10 Gy was found to be equally effective in some but not all studies [6,22,23], and the use of higher cumulative doses of radiation does not provide further benefit [23-25]. Thus, at present the dose of 20 Gy is considered the optimal dose for orbital radiotherapy for GO. The dose is fractionated in 10 daily doses over a 2-week period to reduce the cataractogenic effect of irradiation [26]. Recently, Kahaly et al. [25] showed that 1 Gy/week protocol was better tolerated than the classical 2-week scheme but the longer treatment duration makes this protocol less practical.

Orbital radiotherapy, when properly performed, is usually well tolerated. It may be associated with a transient exacerbation of inflammatory eye signs and symptoms [27] which can be attenuated by steroids [28]. The reported side effects of radiotherapy include cataracts (12% incidence after a median 11 years, radiation retinopathy and second malignancy (calculated risk of 1.2% [29]). In our study, none of the patients developed any of the known adverse events; keeping in mind that long-term follow-up is available only for nine patients. The major concern of physicians and the patients relates to the possibility that orbital radiotherapy may be carcinogenic. No second cancer has been detected yet in our small patient population.

In our series, almost all patients had good outcome immediately after RT. A number of retrospective studies have reported the efficacy of orbital irradiation [30-33]. Most of them noted improvement in soft tissue inflammation but less improvement in motility or amount of proptosis. Donaldson et al. [27] who were the first to use a 4-6MV linear accelerator in a group of 23 patients with severe GO reported good response in 65% of cases, including those who had previously poor response to systemic glucocorticoid treatment. Marcocci [34] summarized the results of 25 publications on radiation for GO and noted that on average, 60% of patients responded favorably to radiation. Prospective studies were also conducted to test the efficacy of radiation in treating GO. Wiersinga et al. [35] reported improvement at 6 months in 64% of patients who received 20 Gy after achieving poor response to steroids or in whom steroids were contraindicated. In another prospective trial [29], patients were randomized to radiation or sham treatment. Improvement was observed in 60% of the radiation group compared with 31% in the sham group. Improvements were seen only in motility (degree of diplopia), but not in soft tissue swelling, which is in contrast to the findings noted in most retrospective series [6]. In our study, 12 patients (71%) received steroids prior to RT and 94% had concurrent steroids with RT with high IV doses. Although radiation provides long-term benefits and a more sustained action for the treatment of GO [21,36], it might take several weeks to show its effects, while corticosteroid pulse therapy produces short-term benefits because of its rapid action. The response rate to steroids in thyroid eye disease varies from 33% to 77% [37-39]. The soft-tissue changes, consisting of edema of the conjunctiva, eyelids, and periorbital area, show the most marked improvement with steroids while the reduction in proptosis and the improvement in ocular motility are usually less impressive [9]. Some evidence suggests that short-term high doses of intravenous steroids are better tolerated by patients and may improve outcome in patients undergoing radiation compared with patients undergoing slow tapering of prednisone [40]. Sustained improvement in visual acuity, eye muscle motility, proptosis, and rectus muscle thickness have also been reported to better respond to IV therapy. This is the reason why the great majority of our patients received high doses of IV steroids during RT. In a study by Bartalena et al [28], patients were randomly assigned to combined therapy with orbital cobalt irradiation and systemic methylprednisolone or to systemic methylprednisolone alone. The treatment responses were less satisfactory with methylprednisolone group compared to the other group. The findings were in accordance with other randomized prospective studies showing that orbital radiotherapy combined with high-dose oral glucocorticoids was more effective in reducing soft tissue changes and ocular motility than orbital radiotherapy alone [41,42]. In contrast, another nonrandomized clinical trial [43] showed that high-dose IV methylprednisolone pulse therapy alone was as effective as high-dose IV methylprednisolone pulse therapy followed by orbital radiotherapy in reducing muscle hypertrophy in Japanese patients with GO. However, no beneficial therapeutic effect on proptosis was observed in that study in either group at 1 month and 6 months of treatment.

## Conclusion

In this retrospective study, we were able to show that combined therapy with RT and high dose steroids is a good option for patients with TAO even in those who have already failed previous treatment with steroids alone or decompression surgery. This treatment modality is well tolerated and long-term complications are almost nonexisting.

## Competing interests

There is no conflict of interest that could be perceived as prejudicing the impartiality of our study.

## Authors' contributions

All authors read and approved the final manuscript.

MA wrote the paper, AA, IS and FG edited the manuscript.

## Funding

Our study did not receive any specific grant from any funding agency in the public, commercial or not-for-profit sector.
